# Interleukin‐36α as a potential biomarker for renal tubular damage induced by dietary phosphate load

**DOI:** 10.1002/2211-5463.12845

**Published:** 2020-04-04

**Authors:** Yoshitaka Hirano, Hiroshi Kurosu, Kazuhiro Shiizaki, Yoshitaka Iwazu, Shuichi Tsuruoka, Makoto Kuro‐o

**Affiliations:** ^1^ Division of Anti‐aging Medicine Center for Molecular Medicine Jichi Medical University Shimotsuke Japan; ^2^ Department of Nephrology Graduate School of Medicine Nippon Medical School Tokyo Japan; ^3^ Department of Clinical Laboratory Medicine Jichi Medical University Shimotsuke Japan; ^4^ AMED‐CREST Japan Agency for Medical Research and Development Tokyo Japan

**Keywords:** dietary phosphate load, interleukin‐36, phosphate excretion per nephron, renal tubular damage

## Abstract

Excessive intake of phosphate has been known to induce renal tubular damage and interstitial inflammation, leading to acute kidney injury or chronic kidney disease in rodents and humans. However, sensitive and early biomarkers for phosphate‐induced kidney damage remain to be identified. Our previous RNA sequencing analysis of renal gene expression identified interleukin‐36α (IL‐36α) as a gene significantly upregulated by dietary phosphate load in mice. To determine the time course and dose dependency of renal IL‐36α expression induced by dietary phosphate load, we placed mice with or without uninephrectomy on a diet containing either 0.35%, 1.0%, 1.5%, or 2.0% inorganic phosphate for 10 days, 4 weeks, or 8 weeks and evaluated renal expression of IL‐36α and other markers of tubular damage and inflammation by quantitative RT‐PCR, immunoblot analysis, and immunohistochemistry. We found that IL‐36α expression was induced in distal convoluted tubules and correlated with phosphate excretion per nephron. The increase in IL‐36α expression was simultaneous with but more robust in amplitude than the increase in tubular damage markers such as Osteopontin and neutrophil gelatinase‐associated lipocalin, preceding the increase in expression of other inflammatory cytokines, including transforming growth factor‐α, interleukin‐1β, and transforming growth factor‐β1. We conclude that IL‐36α serves as a marker that reflects the degree of phosphate load excreted per nephron and of associated kidney damage.

## Graphical abstract

An increase in dietary phosphate intake triggers renal tubular damage, followed by interstitial inflammation and fibrosis. Among several molecular markers indicative of these pathological changes, we identified induction of interleukin‐36α expression in distal tubules as an early and robust biomarker of phosphate‐induced tubular damage in mice.

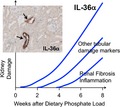

**Abbreviations**
AKI: Acute kidney injury
Ccr7: C‐C chemokine receptor type 7
Cd11c: cluster of differentiation 11c
CKD: chronic kidney disease
Hepes: 4‐(2‐hydroxyethyl)‐1‐piperazineethanesulfonic acid
IL‐1β: interleukin‐1β
IL‐1RAcP: interleukin‐1 receptor accessory protein
IL‐36α: interleukin‐36α
IL‐36R: interleukin‐36 receptor
IRI: ischemia–reperfusion injury
Kim‐1: kidney injury molecule‐1
Mcp‐1: monocyte chemotactic protein‐1
NFκB: nuclear factor‐kappa B
Ngal: neutrophil gelatinase‐associated lipocalin
Nlrp3: nucleotide‐binding oligomerization domain‐like receptor family, pyrin domain‐containing 3
OSP: oral sodium phosphate tablets
Tgf‐β1: transforming growth factor‐β1
Tnf‐α: transforming growth factor‐α
UNx: uninephrectomy
UUO: unilateral ureteral obstruction

Phosphorus is an essential element of life both structurally and functionally [1]. It is a major constituent of the skeleton, cell membrane, and DNA/RNA in the form of calcium phosphate, phospholipids, and nucleic acids, respectively. It also participates in the regulation of energy metabolism and cell signaling in the form of high‐energy phosphate bonds and kinase‐mediated protein phosphorylation. Although indispensable for life, phosphorus can be harmful when ingested excessively. In humans, administration of oral sodium phosphate tablets (OSP) for colon cleansing before colonoscopy occasionally results in acute kidney injury (AKI) at the frequency of 1–4% with increased risk in the aged and in patients with chronic kidney disease (CKD) [2]. In rodents, high‐phosphate diet has been often used in combination with uninephrectomy (UNx) and subtotal nephrectomy to accelerate and enhance kidney damage [3]. In response to increased phosphate intake, urinary phosphate excretion must be increased to maintain the phosphate homeostasis. This demand is met by increasing phosphate excretion per nephron, which was reported to correlate with renal tubular damage and interstitial inflammation [4]. This is consistent with the finding in humans that the risk for OSP‐induced AKI was increased in the aged and in CKD patients whose functional nephron number is assumed to be low [2]. Thus, not only the amount of phosphate intake but also the number of functional nephrons determines the severity of kidney damage induced by dietary phosphate load. However, the mechanism by which the increase in phosphate excretion per nephron induces kidney damage has not been clarified.

To obtain a clue to understand the mechanism, we previously performed RNA sequencing analysis using kidneys from uninephrectomized mice fed the high‐phosphate diet (2.0% inorganic phosphate) and from sham‐operated mice fed the regular diet (0.35% inorganic phosphate) [5]. Besides expression of markers for renal tubular damage including Osteopontin, neutrophil gelatinase‐associated lipocalin (Ngal), and kidney injury molecule‐1 (Kim‐1), expression of several inflammatory cytokines and chemokines was upregulated, suggesting that inflammation might play a critical role in the phosphate‐induced kidney damage. In the present study, we report a unique expression profile of IL‐36α during chronic dietary phosphate load in mice and discuss the potential role that it may play in the pathophysiology and usefulness as a biomarker for phosphate‐induced kidney damage.

## Materials and methods

### Mice

All animal experiments were approved by the Institutional Animal Care and Use Committee from Jichi Medical University (approval number 18003‐01). Male C57BL/6J mice at 8 weeks of age were subjected to either right UNx or sham operation (laparotomy alone) as previously described [5]. Four weeks after the surgery, the mice were placed on a diet containing 0.35%, 1.0%, 1.5%, or 2.0% inorganic phosphate for 10 days. Alternatively, male C57BL/6J mice at 12 weeks of age were placed on a diet containing 0.35% or 2.0% inorganic phosphate for 4 or 8 weeks. The mice were euthanized to harvest the left kidneys. A part of the kidney was snap‐frozen in liquid nitrogen and stored at −80 °C until used for RNA and protein extraction. The rest of the kidney was subjected to histological and immunohistochemical analyses as described below.

### Histological and immunohistochemical analyses

After removing renal capsule, kidneys were fixed in 10% formalin solution overnight and then embedded in paraffin wax. Transverse sections of 3 μm thickness were subjected to von Kossa staining to detect calcium phosphate deposits or immunohistochemical staining of IL‐36α, Klotho, F4/80, and Osteopontin protein. Briefly, the sections were deparaffinized in xylene and rehydrated in a graded EtOH series. Endogenous peroxidase activity was blocked with 3% hydrogen peroxide in distilled water. For antigen retrieval, the sections were boiled in Target Retrieval Solution (Dako, Agilent Pathology Solutions, Santa Clara, CA, USA) for 15 min. The sections were blocked with PBS containing 0. 2% (v/v) Triton X‐100, 1% BSA, and 5% rabbit serum (IL‐36a and Klotho) or 5% goat serum (F4/80), or a blocking reagent included in Histofine Simple Stain Mouse MAX PO (Nichirei Corporation, Tokyo, Japan) (Osteopontin). After blocking, the sections were incubated with a goat polyclonal antibody against mouse IL‐36α (diluted 1 : 100 with PBS containing 1% BSA; AF2297; R&D Systems, Minneapolis, MN, USA), a goat polyclonal antibody against mouse Klotho (diluted 1 : 100 with PBS containing 1% BSA; BAF1819; R&D Systems), a rat monoclonal antibody against mouse F4/80 (diluted 1 : 100 with PBS containing 1% BSA; GTX26640; GeneTex, San Antonio, TX, USA), or a mouse monoclonal antibody against mouse Osteopontin (diluted 1 : 100 with PBS containing 1% BSA; sc‐21742; Santa Cruz, Dallas, TX, USA) at 4 °C overnight and then with Histofine Simple Stain Mouse MAX PO (Nichirei Corporation) at room temperature for 1 h. Immunoreactivity was visualized by incubation with the DAB substrate kit (Dako; Agilent Pathology Solutions). The sections were counterstained with hematoxylin. Images of the stained sections were digitized and analyzed using a microscope (BX‐51; Olympus, Tokyo, Japan).

### Quantification of histological findings

Using computerized image analysis software (image pro 9.32; Media Cybernetics, Rockville, MD, USA), we quantified the collagen volume fraction stained with Picro‐Sirius Red and the calcification volume fraction stained with von Kossa in cortical tubulointerstitial area. We also quantified F4/80 expression in cortical tubules as the percentage of antibody‐positive areas within a given microscope field in cortical tubulointerstitial areas without vessels or glomeruli. Data were expressed as the percentage of the total tubulointerstitial field area. In each sample, at least five randomly selected cortical tubulointerstitial fields (0.162 mm^2^ per field) were assessed at ×400 magnification for quantification of collagen volume fraction and F4/80‐positive areas, and a single kidney section (16.2 mm^2^ per field) was assessed at ×40 magnification for quantification of calcification volume fraction. Quantification was performed in a blinded manner.

### Immunoblot analysis

The frozen kidneys were crushed by Cryo‐Press (Microtec Co., LTD, Chiba, Japan). The tissue powder was homogenized in lysis buffer containing 25 mm Na‐Hepes (pH 7.4), 100 mm NaCl, 0.75% (v/v) NP‐40, 1 mm EDTA, and 1× protease inhibitor cocktail (cOmplete; Roche, Mannheim, Germany). After severing genomic DNA by sonication, the lysate containing 15 μg protein was separated by SDS/PAGE and probed with the antibody against mouse IL‐36α (AF2297; R&D Systems) or actin (MAB1501, clone C4; Millipore, Darmstadt, Germany).

### Quantitative RT‐PCR (qPCR)

The frozen kidneys were homogenized in RNAiso Plus (Takara, Tokyo, Japan). The lysate was transferred to microcentrifuge tube and extracted with one‐fifth volume of chloroform. RNA in the aqueous phase was precipitated with an equal volume of isopropanol, washed with 75% EtOH, and dissolved in RNase‐free water. Reverse transcription of RNA (0.5 μg) was carried out using ReverTra Ace qPCR RT Master Mix with gDNA Remover (FSQ‐301; Toyobo, Osaka, Japan) according to the manufacturer’s protocol. Quantitative RT‐PCRs contained 12.5 ng of cDNA, 410 nm of each primer, and 6 μL of SYBR Green PCR Master Mix (THUNDERBIRD SYBR qPCR Mix, QPS‐201; Toyobo) in a total volume of 12 μL. The reaction (95 °C for 1 min followed by 45 cycles of 95 °C for 10 s, 60 °C for 50 s) was performed on the Roche LC480 system. Relative mRNA levels were calculated by the comparative threshold cycle method using cyclophilin as an internal control. The forward (F) primers and reverse (R) primers used were as follows:

Cyclophilin, F: TGGAGAGCACCAAGACAGACA, R: TGCCGGAGTCGACAATGAT;

IL‐36α, F: CAGCATCACCTTCGCTTAGAC, R: AGTGTCCAGATATTGGCATGG;

nucleotide‐binding oligomerization domain‐like receptor family, pyrin domain‐containing 3 (Nlrp3), F: CGAGACCTCTGGGAAAAAGCT, R: GCATACCATAGAGGAATGTGATGTACA;

Ngal, F: GAAATATGCACAGGTATCCTC, R: GTAATTTTGAAGTATTGCTTGTTT;

Osteopontin, F: TCCAAAGAGAGCCAGGAGAG, R: GGCTTTGGAACTTGCTTGAC;

monocyte chemotactic protein‐1 (Mcp‐1), F: GGCTCAGCCAGATGCAGTTAAC, R: GCCTACTCATTGGGATCATCTTG;

Kim‐1, F: CTGGAATGGCACTGTGACATCC, R: GCAGATGCCAACATAGAAGCCC;

transforming growth factor‐α (Tnf‐α), F: AAGCCTGTAGCCCACGTCGTA, R: GGCACCACTAGTTGGTTGTCTTTG;

interleukin‐1β (IL‐1β), F: TGAAGTTGACGGACCCCAAA, R: TGATGTGCTGCTGCGAGATT;

transforming growth factor‐β1 (Tgf‐β1), F: TTGCTTCAGCTCCACAGAGA, R: TGGTTGTAGAGGGCAAGGAC;

cluster of differentiation 11c (Cd11c), F: GTTTGTCTCAGACGGG, R: GCGGGTTCAAAGACGATGG; and

C‐C chemokine receptor type 7 (Ccr7), F: CTCTTCAAGGACTTGGGCTG, R: CCTGGGAGAGGTCCTTGTAG.

### Statistics

Data were analyzed using the Wilcoxon rank‐sum test, or the Kruskal–Wallis test, followed by *post hoc* analysis with the Steel–Dwass test, where appropriate. A *P*‐value < 0.05 was considered statistically significant.

## Results

**Fig. 1:**
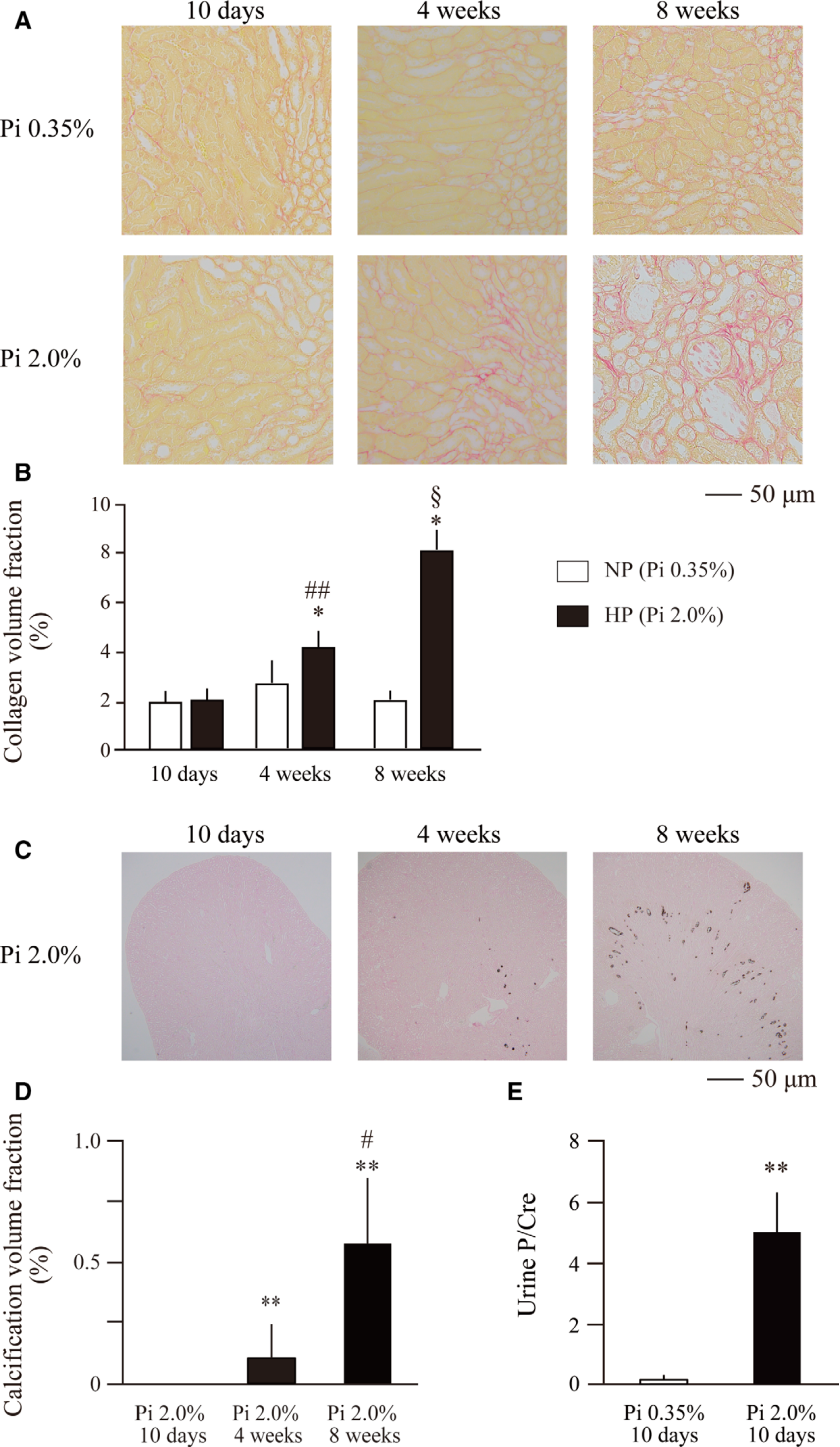
Fibrosis and nephrocalcinosis induced by dietary phosphate load in mice. Wild‐type mice at 12 weeks of age were placed on the regular diet (Pi 0.35%, NP) or high‐phosphate diet (Pi 2.0%, HP) for 10 days (*N* = 8 for Pi 0.35%, *N* = 8 for Pi 2.0%), 4 weeks (*N* = 7 for Pi 0.35%, *N* = 8 for Pi 2.0%), and 8 weeks (*N* = 5 for Pi 0.35%, *N* = 5 for Pi 2.0%), respectively. (A) Sirius Red staining detected interstitial fibrosis in the kidneys. Bar = 50 μm. (B) The area of the Sirius Red‐positive regions was measured and expressed as a percentage of the total area using image pro Version 9.32. The data are presented as mean ± SD. *P < 0.05 vs mice on the high‐phosphate diet for 10 days, ##*P* < 0.01 vs mice on the high‐phosphate diet for 10 days, and §*P* < 0.05 vs mice on the high‐phosphate diet for 4 weeks by the Steel–Dwass test. (C) von Kossa staining of paraffin‐embedded kidney sections in mice fed a high‐phosphate diet (2.0% Pi) for 10 days (*N* = 8), 4 weeks (*N* = 6), and 8 weeks (*N* = 5). Bar = 50 μm. (D) The area of the von Kossa‐positive regions was measured and expressed as a percentage of the total area using image pro Version 9.32. The data are presented as mean ± SD. **P < 0.01 vs mice on the high‐phosphate diet for 10 days and #*P* < 0.05 vs mice on the high‐phosphate diet for 4 weeks by the Steel–Dwass test. (E) Urine phosphate concentration corrected with urine creatinine concentration in mice fed the high‐phosphate diet (Pi 2.0%: solid bars) and normal phosphate diet (Pi 0.35%: empty bars) for 10 days (*N* = *8* per each group). The data are presented as mean ± SD. **P < 0.01 vs mice on the normal phosphate diet for 10 days by the Steel–Dwass test.

**Fig. 2:**
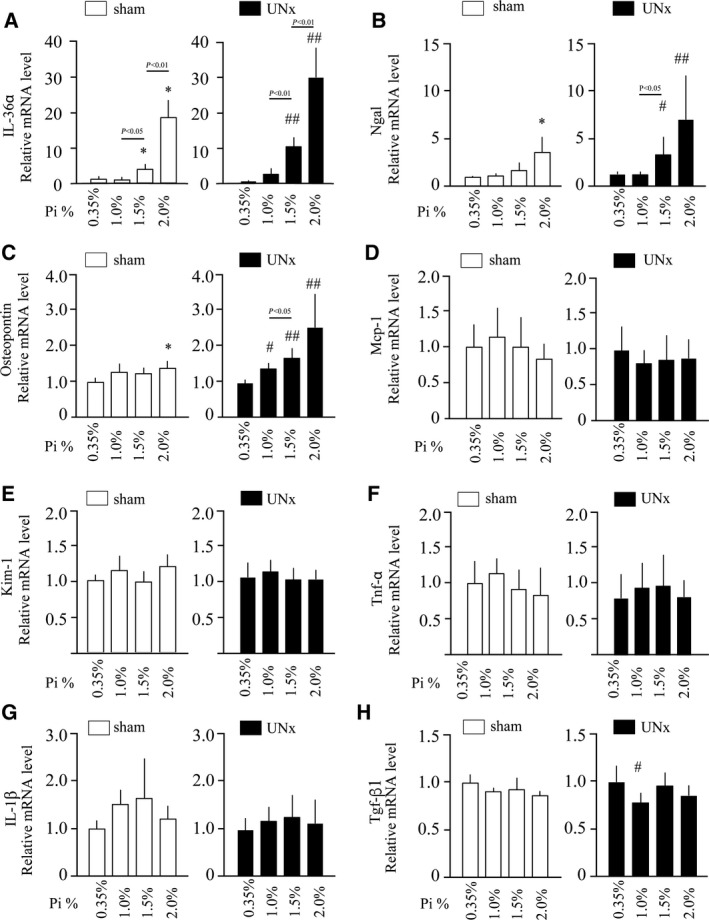
Effects of dietary phosphate load on expression of markers for renal tubular damage and inflammation in mice with or without UNx. Wild‐type mice were subjected to either sham operation (Sham; empty bars) or UNx (solid bars) followed by a diet containing either 0.35%, 1.0%, 1.5%, or 2.0% inorganic phosphate (Pi) for 10 days. Relative mRNA expression levels of interleukin‐36α (IL‐36α) (A), Ngal (B), Osteopontin (C), Mcp‐1 (D), Kim‐1 (E), Tnf‐α (F), interleukin‐1β (IL‐1β) (G), and Tgf‐β1 (H) in the kidney are indicated. All data are presented as mean ± SD. All statistical analysis was performed by the Steel–Dwass test: *P < 0.05 vs 0.35%Pi Sham, #*P* < 0.05 vs 0.35%Pi UNx, and ##*P* < 0.01 vs 0.35%Pi UNx. The number of sham‐operated mice fed the diet containing 0.35%, 1.0%, 1.5%, or 2.0% inorganic phosphate was 6, 8, 7, and 8, respectively. The number of UNx mice fed the diet containing 0.35%, 1.0%, 1.5%, or 2.0% inorganic phosphate was 7, 7, 8, and 8, respectively.

When normal adult mice were placed on a high‐phosphate diet containing 2.0% inorganic phosphate, depositions of collagen and calcium phosphate became detectable within 4 weeks in renal tubules around the cortico‐medullary junction (CMJ), which progressed to overt tubulointerstitial fibrosis and nephrocalcinosis within 8 weeks (Fig. 1A–D). Because an increase in urinary phosphate excretion was observed much earlier (Fig 1E), we speculated that exposure of renal tubular cells to high‐phosphate tubular fluid might have caused kidney damage prior to the development of tubulointerstitial fibrosis and nephrocalcinosis. To identify earlier changes that might have occurred before tubulointerstitial fibrosis and nephrocalcinosis became evident at the histological level, we evaluated renal expression of several markers for renal tubular damage (Osteopontin, Ngal, and Kim‐1) and inflammation (Mcp‐1, Tnf‐α, IL‐1β, Tgf‐β1, and IL‐36α) at 10 days after increasing phosphate excretion per nephron (Fig. 2).

We manipulated phosphate excretion per nephron by placing uninephrectomized mice and sham‐operated mice on diet containing either 0.35%, 1.0%, 1.5%, or 2.0% inorganic phosphate for 10 days. Phosphate excretion per nephron in the uninephrectomized mice was supposed to be twofold higher than that in the sham‐operated mice placed on the same diet. Quantitative RT‐PCR (qPCR) analysis revealed significant upregulation of Ngal (Fig. 2B) and Osteopontin (Fig. 2C), but not Kim‐1 (Fig. 2E), with an increase in the dietary phosphate content. Despite the evidence for renal tubular damage, histological examination failed to detect collagen and calcium phosphate deposits within 10 days after the phosphate load, indicating that renal tubular damage at the molecular level had started much earlier than development of tubulointerstitial fibrosis and nephrocalcinosis at the histological level. Notably, uninephrectomized mice showed higher Osteopontin and Ngal mRNA levels than sham‐operated mice placed on the same diet (1.5% and 2.0% inorganic phosphate). This finding is consistent with the previous observation that phosphate excretion per nephron correlated with renal tubular damage [4]. In contrast to the markers for tubular damage, those for inflammation were not upregulated at this time point (Fig. 2D,F,G,H) except IL‐36α (Fig. 2A). Renal IL‐36α expression was undetectable in mice fed the regular diet (0.35% inorganic phosphate) but significantly upregulated within 10 days after starting dietary phosphate load in a dose‐dependent manner. Like Osteopontin and Ngal, IL‐36α mRNA levels were higher in uninephrectomized mice than those in sham‐operated mice fed the same diet (1.5% and 2.0% inorganic phosphate).

**Fig. 3:**
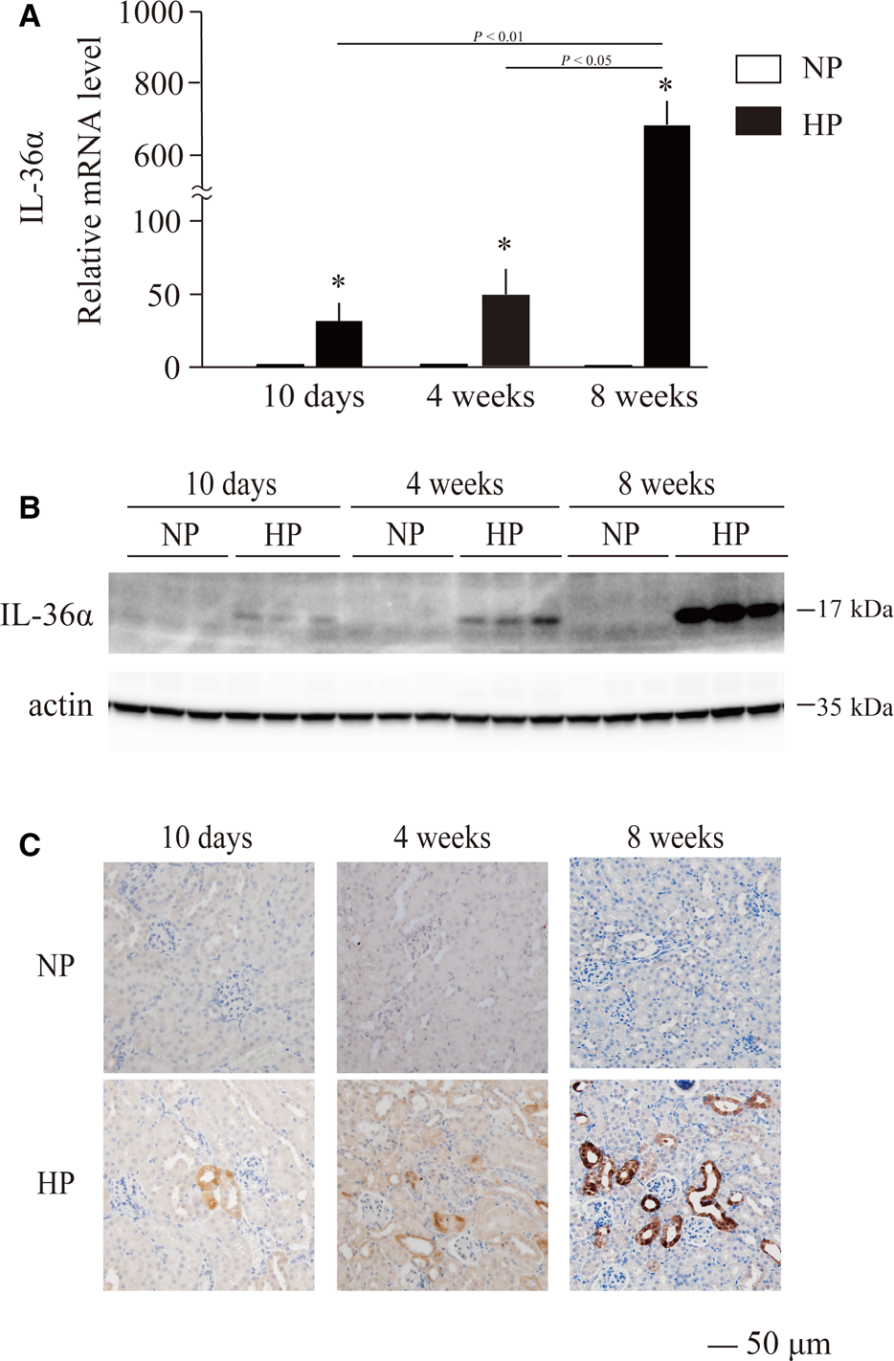
Effects of prolonged dietary phosphate load on renal IL‐36α expression. Wild‐type mice at 12 weeks of age were placed on a regular diet containing 0.35% Pi (NP) or a high‐phosphate diet containing 2.0% Pi (HP) for 10 days (*N* = 8), 4 weeks (*N* = 6), or 8 weeks (*N* = 5). Kidneys were excised from mice fed a diet containing 0.35% and 2.0% inorganic phosphate for 10 days, 4 weeks, and 8 weeks. (A) Relative mRNA expression levels of IL‐36α in the kidney are indicated. All data are presented as mean ± SD. *P < 0.01 vs mice 0.35% Pi‐fed mice, by Wilcoxon’s rank‐sum test. (B) Immunoblot analysis of the kidney lysates using the antibody against IL‐36α and β‐actin as an internal control. (C) Immunohistochemical staining for IL‐36α protein in the in kidney from mice fed the NP or HP for 10 days, 4 weeks, and 8 weeks. Bar = 50 μm.

**Fig. 4:**
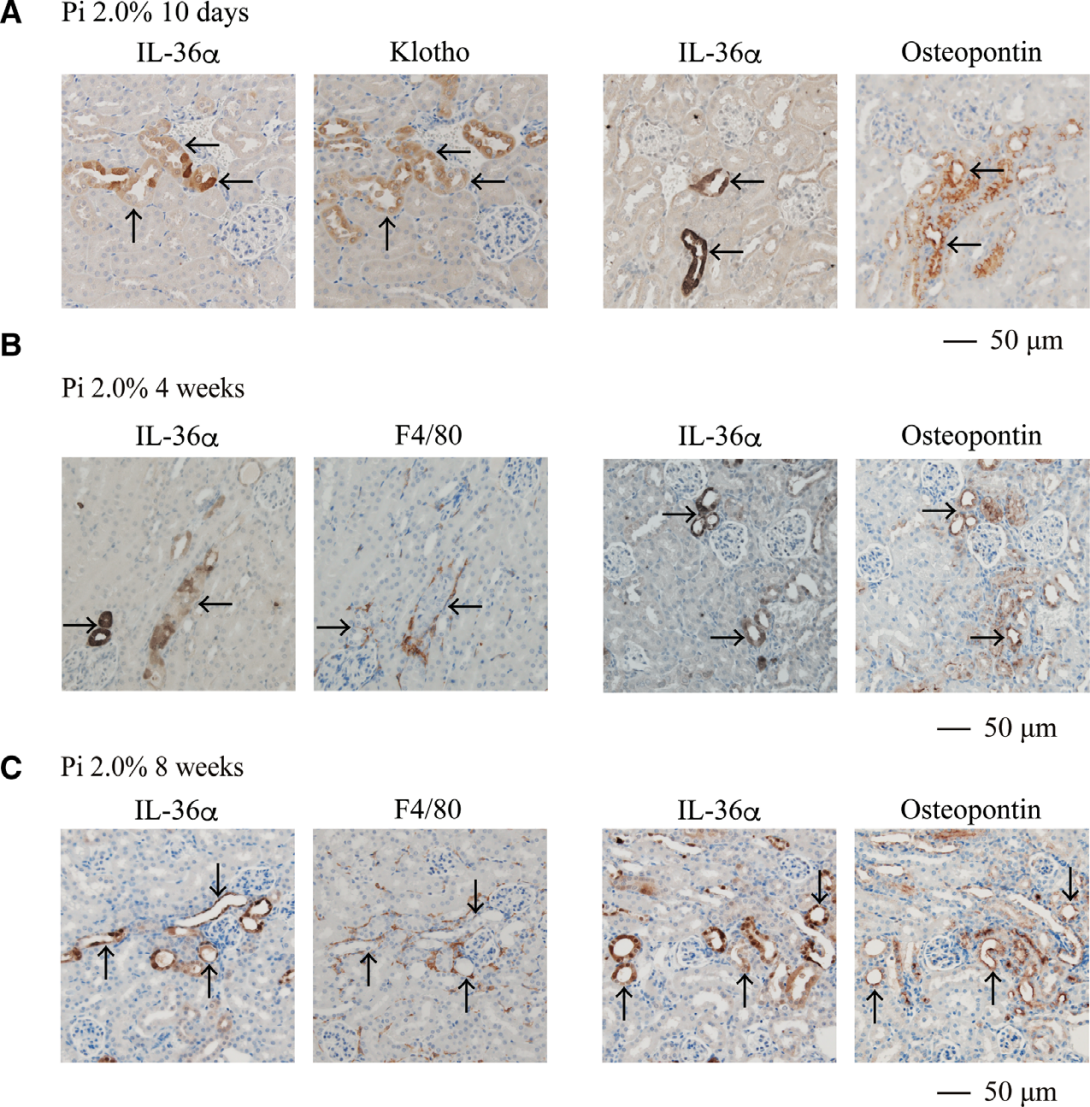
Localization of IL‐36α expression induced by phosphate load. Wild‐type mice at 12 weeks of age were placed on a high‐phosphate diet containing 2.0% Pi for 10 days (A), 4 weeks (B), and 8 weeks (C). Serial paraffin sections of the kidneys were subjected to immunohistochemical staining using antibodies against IL‐36α, Klotho, Osteopontin, and F4/80. (A) Klotho‐positive tubules expressed IL‐36α. Osteopontin‐positive tubules expressed IL‐36α. (B, C) IL‐36α‐positive tubules expressed Osteopontin and were surrounded by F4/80‐positive cells. Arrows indicate identical renal tubule in serial sections. Bar = 50 μm.

Renal expression of IL‐36α was further increased after prolonged dietary phosphate load. Normal adult mice placed on high‐phosphate diet containing 2.0% inorganic phosphate had 47.9‐fold and 697.8‐fold higher IL‐36α mRNA levels within 4 and 8 weeks, respectively, than mice placed on a regular diet containing 0.35% inorganic phosphate (Fig. 3A). Consistent with the mRNA levels, IL‐36α protein was detectable by immunoblot analysis in the kidney lysates from mice fed the high‐phosphate diet for 4 and 8 weeks, but not in those from mice fed the regular diet (Fig. 3B). In immunohistochemical analysis, strong IL‐36α expression was observed specifically in some renal tubules in the cortex (Fig. 3C), which did not colocalize with calcium phosphate depositions that appeared mainly in the CMJ but colocalized with Klotho, which is expressed in distal convoluted tubules [6] (Fig. 4A, arrows). IL‐36α‐positive tubular cells also expressed Osteopontin, indicating that IL‐36α is expressed in injured renal tubules. After prolonged dietary phosphate load (4 and 8 weeks), Klotho expression was downregulated and difficult to detect by immunohistochemistry (data not shown). However, we confirmed consistent colocalization of IL‐36α and Osteopontin in renal tubular cells throughout the observation period (Fig. 4, arrows). Although F4/80‐positive macrophages were infiltrated around the IL‐36α‐positive tubules (Fig. 4B,C), they were negative for IL‐36α, suggesting that IL‐36α expression may be basically restricted to damaged renal tubules.

**Fig. 5:**
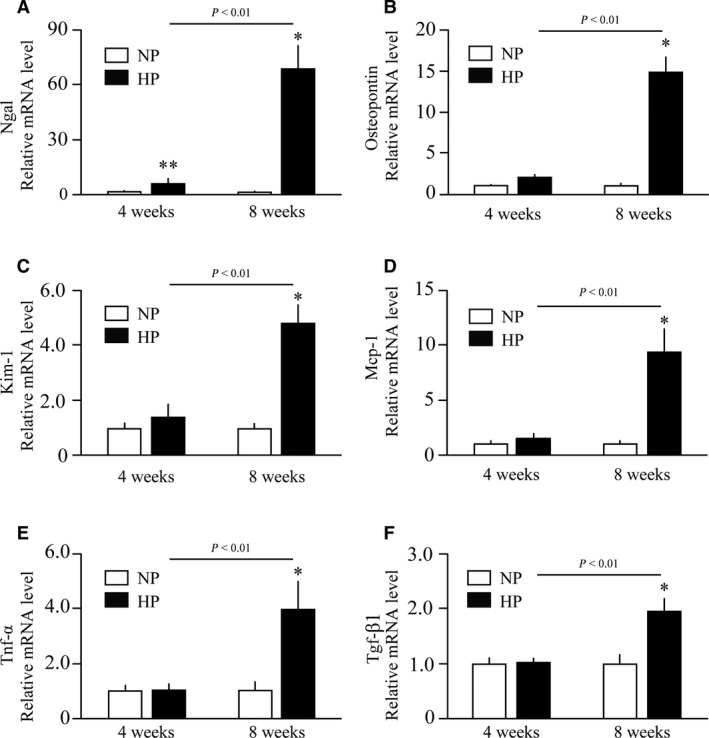
Effects of prolonged dietary phosphate load on renal expression of markers for renal tubular damage and inflammation. Wild‐type mice at 12 weeks of age were placed on a regular diet containing 0.35% Pi (NP) or 2.0% Pi (HP) for 4 weeks (*N* = 6 per group) or 8 weeks (*N* = 5 per group). Relative mRNA levels of Ngal (A), Osteopontin (B), Kim‐1 (C), Mcp‐1 (D), tumor necrosis factor‐α (Tnf‐α) (E), and Tgf‐β1 (F) are indicated. All data are presented as mean ± SD. *P < 0.05 vs mice fed 0.35%Pi for 8 weeks, **P < 0.01 vs mice fed 0.35%Pi for 4 weeks by Wilcoxon’s rank‐sum test.

**Fig. 6:**
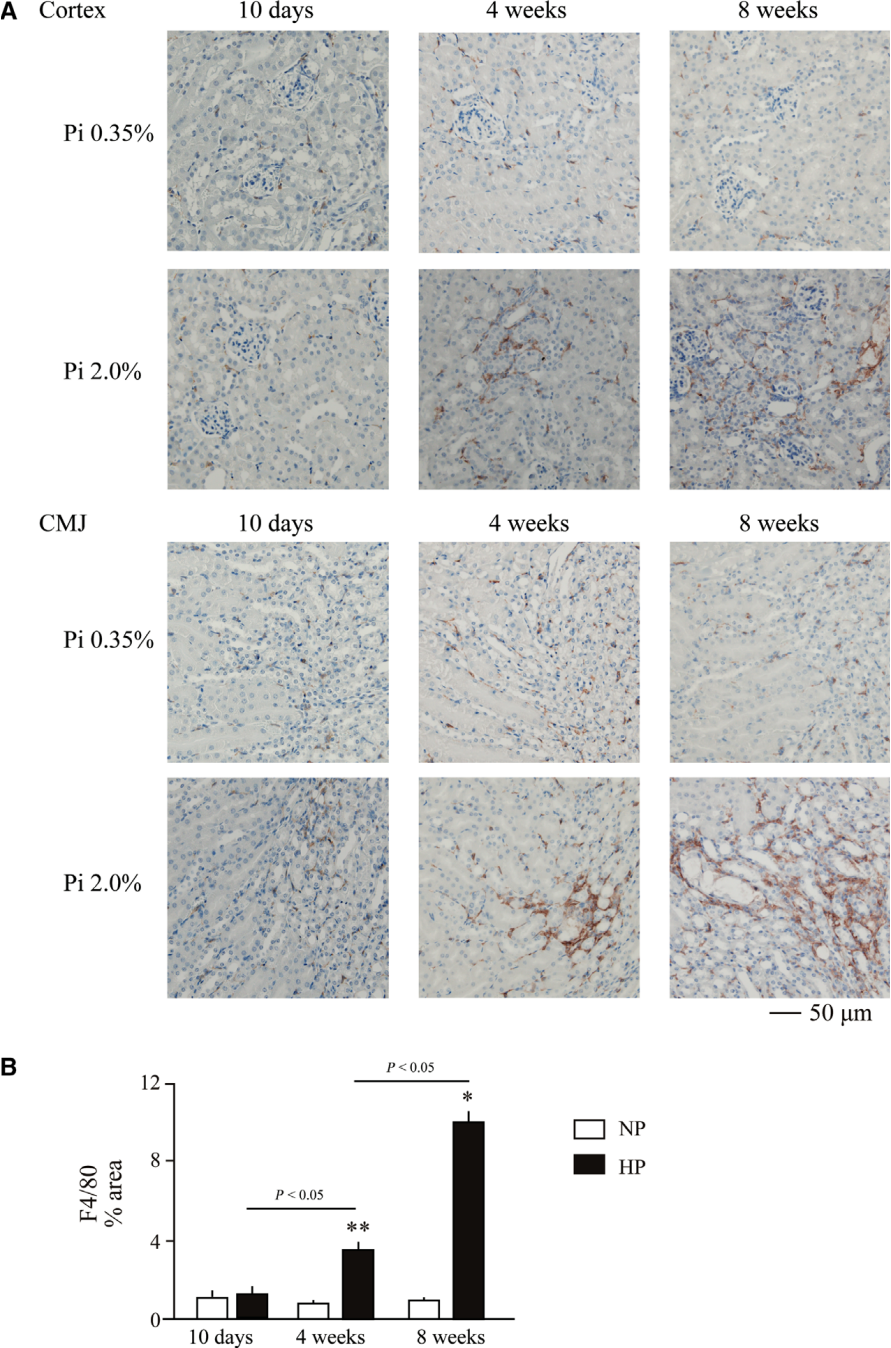
Interstitial macrophage infiltration induced by dietary phosphate load. Wild‐type mice at 12 weeks of age were placed on a regular diet (Pi 0.35%) or high‐phosphate diet (Pi 2.0%) for 10 days, 4 weeks, or 8 weeks. (A) Paraffin sections of the kidneys were subjected to immunohistochemical staining using antibodies against F4/80. Interstitial infiltration of macrophages was observed in the cortex and CMJ in mice fed the high‐phosphate diet for 4 and 8 weeks. Bar = 50 μm. (B) The area of the F4/80‐positive regions was measured and expressed as a percentage of the total area using image pro Version 9.32. Empty and solid bars represent mice fed the regular diet (Pi 0.35%) or high‐phosphate diet (Pi 2.0%), respectively, for 10 days (*N* = 8 for Pi 0.35%, *N* = 8 for Pi 2.0%), 4 weeks (*N* = 6 for Pi 0.35%, *N* = 5 for Pi 2.0%), and 8 weeks (*N* = 5 for Pi 0.35%, *N* = 5 for Pi 2.0%). The data are presented as mean ± SD. The positive area was larger in mice fed the high‐phosphate diet when compared with mice on the regular diet for 4 and 8 weeks. *P < 0.05 vs mice fed 0.35%Pi for 8 weeks and **P < 0.01 vs mice fed 0.35%Pi for 4 weeks, by Wilcoxon’s rank‐sum test.

**Fig. 7:**
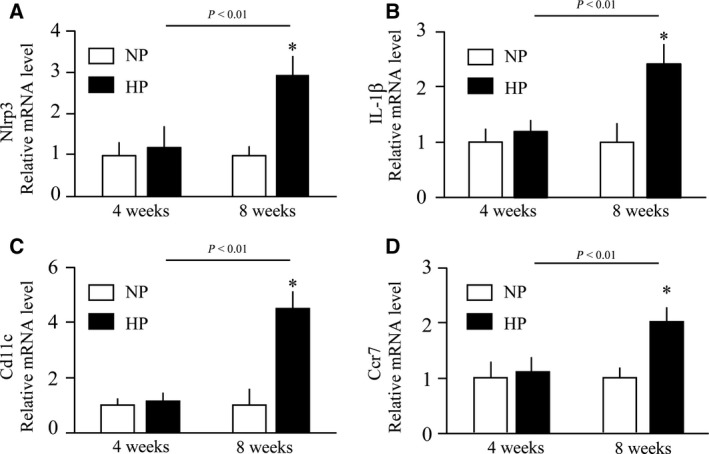
Effects of prolonged dietary phosphate load on renal expression of markers for renal inflammasome and dendritic cells. Wild‐type mice at 12 weeks of age were placed on a regular diet containing 0.35% Pi (NP) or 2.0% Pi (HP) for 4 weeks (*N* = 6 per group) or 8 weeks (*N* = 5 per group). Relative mRNA levels of Nod‐like receptor protein 3 (Nlrp3) (A), IL‐1β (B), Cd11c (C), and Ccr7 (D) are indicated. All data are presented as mean ± SD. *P < 0.05 vs mice fed 0.35% Pi for 4 weeks by Wilcoxon’s rank‐sum test.

Besides IL‐36α, feeding of the high‐phosphate diet for 4 weeks slightly raised the mRNA levels of Ngal (Fig. 5). Interstitial infiltration of F4/80‐positive macrophages was observed in the cortex and CMJ, which was exacerbated with an increase in the period of dietary phosphate load (Fig. 6). After 8 weeks of high‐phosphate diet feeding, expression of all the markers for inflammation and renal tubular damage so far examined was significantly increased (Fig. 5). Of note, expression of Nlrp3 and IL‐1β was also induced (Fig. 7A,B), which are downstream targets of the nuclear factor‐kappa B (NFκB) signaling activated by IL‐36α [7]. Furthermore, mRNA levels of Cd11c and Ccr7 expressed in dendritic cells [8] were increased (Fig. 7C,D), indicating dendritic cell infiltration that was reported to be induced by IL‐36α [9].

## Discussion

The present study identified IL‐36α as a robust and early marker for kidney damage induced by increased phosphate excretion per nephron. IL‐36α is a recently identified pro‐inflammatory cytokine that belongs to the IL‐1 family and reported to contribute to the pathophysiology of several disorders associated with inflammation, including psoriasis, chronic obstructive pulmonary disease, and obesity [10, 11]. Like other cytokines, IL‐36α is expressed primarily in immuno‐competent cells, but is induced in renal tubular cells in patients and rodent models with AKI and CKD [9, 12]. In mice, unilateral ureteral obstruction (UUO), ischemia–reperfusion injury (IRI), glomerulonephritis, and diabetic nephropathy were reported to induce expression of IL‐36α in renal tubular cells [9, 13, 14]. In humans, urine levels of IL‐36α were reported to increase in patients with AKI [12] and CKD [9]. These findings suggest that IL‐36α may serve as a biomarker for various types of kidney damage.

It is possible to argue that calcium phosphate precipitates might cause tubular obstruction, which potentially contributed to the tubular damage and thus increased IL‐36α expression. In fact, tubular dilatation was observed in mice fed the high‐phosphate diet for 4 weeks or longer (Figs 1A and 3C). However, this does not explain the early phase of IL‐36α induction that occurred before calcium phosphate precipitations became detectable at the histological level at 4 weeks of phosphate load. These findings suggest that phosphate itself, rather than tubular obstruction caused by calcium phosphate precipitations, may play a pathogenic role at least in the early phase during the course of tubular damage progression caused by dietary phosphate load, although the mechanism remains to be determined.

Osteopontin, Ngal, and Kim‐1 were used as markers for renal tubular damage in this study. Osteopontin is a secreted phosphoglycoprotein with multiple functions, including recruitment of macrophages [15] and inhibition of calcium phosphate nucleation and aggregation [16]. Osteopontin appears protective against calcification, because mice lacking Osteopontin were prone to nephrocalcinosis and stone formation [16]. Thus, induction of Osteopontin expression by dietary phosphate load can be regarded as a defense mechanism against nephrocalcinosis. Ngal is an early and robust marker for renal tubular damage reported to rise in urine about 1000‐fold within hours in a mouse IRI model [17] and about 20‐fold in patients after cardiac surgery [18]. Although induction of Ngal expression by dietary phosphate load occurred within 10 days and coincided with induction of IL‐36α (Fig. 2A,B), the maximum induction observed in the uninephrectomized mice fed 2.0% phosphate diet was no more than sevenfold and less potent than the 18.6‐fold increase in IL‐36α expression. Kim‐1 was less robust than Ngal, because induction of Kim‐1 was not clear within 10 days even in the uninephrectomized mice fed the 2.0% phosphate diet (Fig. 2E), and reached 1.46‐fold and 4.83‐fold at most within 4 and 8 weeks, respectively (Fig. 5C). Taken together, IL‐36α may be potentially advantageous to Osteopontin, Ngal, and Kim‐1 as a marker for kidney damage induced by increased phosphate excretion per nephron.

IL‐36α may serve not merely as a marker for kidney damage but play a critical role in the pathogenesis. Binding of IL‐36α to IL‐36 receptor (IL‐36R) recruits IL‐1 receptor accessory protein (IL‐1RAcP) to form the ternary complex composed of IL‐36α, IL‐36R, and IL‐1RAcP and activate NFκB, mitogen‐activated protein kinase [19], and NLRP3 inflammasome, which promotes maturation and secretion of other inflammatory cytokines including IL‐1β and IL‐18 [9]. Chi *et al. *[9] reported that IL‐36α activated NLRP3 inflammasome in the UUO kidney. In addition, they showed that IL‐36α triggered dendritic cell‐induced T‐cell proliferation and Th17 differentiation and that ablation of the IL‐36R gene or administration of an IL‐36R antagonist alleviated the UUO‐induced kidney damage [9]. Thus, a similar mechanism may be applicable to the kidney damage induced by increased phosphate excretion per nephron as well. In fact, a significant increase in Nlrp3, IL‐1β, and dendritic cell markers (Cd11c and Ccr7) was observed within 8 weeks after starting dietary phosphate load (Fig. 7). Further studies are necessary to verify this hypothesis, including dietary phosphate load in IL‐36R‐deficient mice.

## Conclusions

IL‐36α serves as a marker that reflects the degree of phosphate load excreted per nephron and of associated kidney damage.

## Conflict of interest

The authors declare no conflict of interest.

## Author contributions

YH and HK developed the study concept. YH, HK, KS, and YI carried out experiments and analyzed the results under the supervision of ST and MK. MK obtained funding and drafted the manuscript. All authors have read and approved the manuscript.
